# Transcription Factor: A Powerful Tool to Regulate Biosynthesis of Active Ingredients in *Salvia miltiorrhiza*

**DOI:** 10.3389/fpls.2021.622011

**Published:** 2021-02-24

**Authors:** Sijia Wu, Bo Zhu, Luping Qin, Khalid Rahman, Lei Zhang, Ting Han

**Affiliations:** ^1^School of Pharmacy, Second Military Medical University, Shanghai, China; ^2^School of Pharmacy, Zhejiang Chinese Medical University, Hangzhou, China; ^3^Faculty of Science, School of Pharmacy and Biomolecular Sciences, Liverpool John Moores University, Liverpool, United Kingdom

**Keywords:** *Salvia miltiorrhiza*, transcription factor, biosynthetic regulation, tanshinones, phenolic acids

## Abstract

*Salvia miltiorrhiza* Bunge is a common Chinese herbal medicine, and its major active ingredients are phenolic acids and tanshinones, which are widely used to treat vascular diseases. However, the wild form of *S. miltiorrhiza* possess low levels of these important pharmaceutical agents; thus, improving their levels is an active area of research. Transcription factors, which promote or inhibit the expressions of multiple genes involved in one or more biosynthetic pathways, are powerful tools for controlling gene expression in biosynthesis. Several families of transcription factors have been reported to participate in regulating phenolic acid and tanshinone biosynthesis and influence their accumulation. This review summarizes the current status in this field, with focus on the transcription factors which have been identified in recent years and their functions in the biosynthetic regulation of phenolic acids and tanshinones. Otherwise, the new insight for further research is provided. Finally, the application of the biosynthetic regulation of active ingredients by the transcription factors in *S. miltiorrhiza* are discussed, and new insights for future research are explored.

## Introduction

*Salvia miltiorrhiza* Bunge is a small genome size plant; thus, it makes it a model medical plant to study (Lu et al., 2020). The main active ingredients of *S. miltiorrhiza* can be divided into two groups: water-soluble phenolic acids and liposoluble diterpenoid tanshinones. Phenolic acids, like rosmarinic acids and salvianolic acids, are antibacterial, anti-oxidative, and antiviral reagents, Wenping et al. (2011), while tanshinones, such as tanshinone I, tanshinone IIA, dihydrotanshnone I, tanshinone IIB, and cryptotanshinone, exhibit antitumor, antioxidant, and anti-inflammatory activities (Zhou et al., 2017).

Not surprisingly, initial investigations of phenolic acid and tanshinone have mainly focused on establishing their biosynthetic pathways. The biosynthetic pathways of phenolic acids and tanshinones in *S. miltiorrhiza* have been studied by overexpressing or inhibiting key enzyme genes (Gao et al., 2009; Kai et al., 2011; Ma et al., 2013). However, this approach has limited efficiency when compared to transcriptional regulation. Transcription factors (TFs) in plants regulate the biological processes through activating or inhibiting one or multiple pathways (Gao et al., 2014). To date, more than 1,300 TFs have been detected in *S. miltiorrhiza* (Wenping et al., 2011; Luo et al., 2014), including WKRYs, bHLHs, MYBs, AP2/ERFs, and so on. However, the regulatory mechanisms of the biosynthesis of the active ingredient in *S. miltiorrhiza* are still poorly understood.

Here we review the biosynthetic pathways of phenolic acids and tanshinones in *S. miltiorrhiza*, with particular focus on the TFs that regulate the pathways, and highlight effective research approaches for improving the active ingredients of medical plants.

## Biosynthetic Pathways of Phenolic Acids and Tanshinones

**FIGURE 1:**
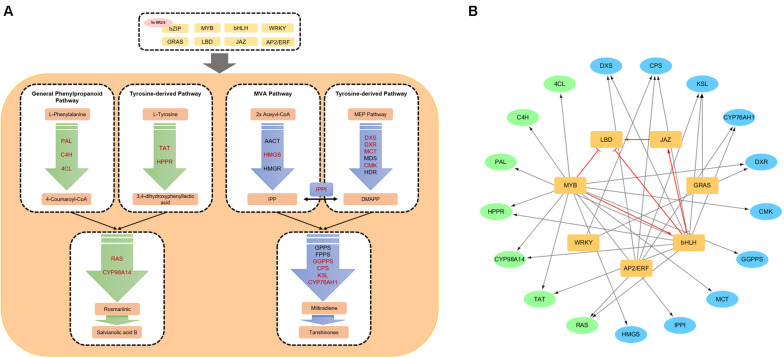
Biosynthetic pathways and a transcriptional regulation network of ingredients in *Salvia miltiorrhiza*. **(A)** A simplified transcriptional regulation network of phenolic acid and tanshinone biosynthetic pathways. Red enzyme genes can be interacted with transcription factors. **(B)** The transcription factor–enzyme gene regulation network. The black arrows indicate the interaction between TFs and enzyme. Red arrows and red T-shaped line indicate upregulation and inhibition between transcription factors.

The simplified biosynthetic pathways of phenolic acids and tanshinones is shown in Figure 1A.

The biosynthetic pathways of the general phenylpropanoid pathway and the tyrosine-derived pathway in *S. miltiorrhiza* have been intensively investigated. The general phenylpropanoid pathway is initiated by the catalytic action of phenylalanine ammonia-lyase (PAL) on the precursor amino acid phenylalanine, which is then catalyzed by cinnamate 4-hydroxylase (C4H) and p-coumaroyl coenzyme A ligase (4CL) into 4-coumaroyl-CoA (Yang et al., 2016). L-Tyrosine is catalyzed by tyrosine aminotransferase (TAT) and 4-hydroxyphenylpyruvate reductase (HPPR) successively into 3,4-dihydroxyphenyllactic acid (Di et al., 2013). 4-Coumaroyl-CoA and 3,4-dihydroxyphenyllactic acid (DHPL) are the two intermediates of the phenolic biosynthetic pathway according to experiments involving [ring-(13)C]-phenylalanine labeling *in vivo*. These are then catalyzed by rosmarinic acid synthase (RAS) to form 4-coumaroyl-3′,4′-dihydroxyphenyllactic acid (4C-DHPL) which is then converted into rosmarinic acid by a P450 monooxygenase, SmCYP98A14 (Di et al., 2013).

All terpenoids are synthesized from sequential assembly of five-carbon building blocks (C5H8) called isoprene units, while the four isoprene units constitute diterpenes (Yang et al., 2016). Isopentenyl diphosphate (IPP) and its isomer dimethylallyl diphosphate (DMAPP) are the two precursors of all terpenoids and are synthesized via two independent pathways: the methylerythritol phosphate (MEP) pathway in the plastids and the mevalonate (MVA) pathway in the cytosol. It was proposed that tanshinones are chiefly synthesized by the MEP pathway rather than the MVA pathway (Ma et al., 2015). Then, geranyl diphosphate synthase (GPPS), farnesyl diphosphate synthase (FPPS), and geranylgeranyl diphosphate synthase (GGPPS) catalyze DMAPP and IPP successively to form geranylgeranyl diphosphate (GGPP), which is the universal precursor of all diterpenoids (Dong et al., 2011). Skeleton miltiradiene in tanshinone biosynthesis is formed from Sm1, SmCPS2, and SmKSL1. In the downstream pathway, P450s participate in tanshinone biosynthesis. Guo et al. (2013) found that a P450 monooxygenase CYP76AH1 transformed miltiradiene to ferruginol. However, reference genes for post-modification characterization involved in biosynthetic pathway need further investigation.

## TFs Regulating Biosynthesis of Phenolic Acid and Tanshinone

In plants, the regulation and accumulation of secondary metabolites is usually controlled by a complex network containing TFs (Yang et al., 2012). And TFs act as switches in regulating secondary metabolites network. The action of TFs possesses three traits: (1) TFs act alone or in a combinatorial fashion with other TFs to modulate the expression of target genes (Pinson et al., 2009; Goossens et al., 2017); (2) TFs can positively or negatively regulate biosynthesis pathways (Table 1); (3) one TF regulates the expression of multiple genes participating in one or more biosynthetic pathways (Goossens et al., 2017; Hassani et al., 2020; Table 1).

**TABLE 1:** TFs positively or negatively regulate ingredients in *S. miltiorrhiza*.

Family	Member	Tanshinone		Phenolic acid		References
		Function	Target	Function	Target	
bHLH	SmbHLH51			Positive		Wu et al., 2018
	SmbHLH10	Positive	DXS2; CPS1; CPS5			Xing et al., 2018b
	SmbHLH148	Positive		Positive		Xing et al., 2018a
	SmbHLH3	Negative	CYP76AH1; KSL1	Negative	TAT; HPPR	Zhang et al., 2020
	SmbHLH37			Negative	TAT1; PAL1	Du et al., 2018
	SmMYC2			Positive	TAT1; PAL1; CYP98A14	Yang et al., 2017
	SmMYC2a	Positive		Positive	CYP98A14; RAS6	Zhou et al., 2016
	SmMYC2b	Positive		Positive	CYP98A14; RAS6	
MYB	SmMYB39			Negative		Zhang et al., 2013
	SmMYB9b	Positive		Negative		Zhang et al., 2017
	SmMYB98	Positive		Positive		Hao et al., 2020
	SmMYB98b	Positive				Liu et al., 2020
	SmMYB111			Positive		Li S. et al., 2018
	SmMYB36	Positive	DXR; MCT; CMK; IPPI; GGPPS1; HMGS1	Negative	C4H1; 4CL1; HPPR1	Ding et al., 2017
	SmMYB2			Positive	CYP98A14	Deng et al., 2020
	MYB1			Positive	CYP98A14	Zhou et al., 2021
ERF	SmERF1L1	Positive	DXS	Negative		Huang et al., 2019
	SmERF115	Negative		Positive	RAS1	Sun et al., 2019
	SmERF6	Positive	CPS1; KSL1	Negative		Bai et al., 2018
	SmERF128	Positive	CYP76AH1; KSL1; CPS1			Zhang et al., 2019
WRKY	SmWRKY2	Positive	CPS			Deng et al., 2019
	SmWRKY1	Positive	DXR			Cao et al., 2018
GRAS	SmGRAS1	Positive	KSL1	Negative		Li et al., 2019
	SmGRAS2	Positive	KSL1	Negative		
	SmGRAS3	Positive	KSL1	Negative		Li et al., 2020
Other	SmAREB1			Positive		Jia et al., 2017
	SmLBD50			Negative		Lu et al., 2020
	SmJAZ8	Negative		Negative		Pei et al., 2018

Currently, several TFs which can regulate phenolic acid and tanshinone biosynthesis have been characterized, and a transcriptional regulation network of ingredients in *S. miltiorrhiza* is shown in Figure 1.

### bHLH Family

The bHLH family is the second largest class of plant TFs (Feller et al., 2011; Goossens et al., 2017) and define their functionality with the specific DNA-4binding domains. The bHLH family harbors two functionally distinct regions in 60 amino acids: the basic region at the N-terminus which can bind to the E-box DNA motif (CANNTG) and the HLH motif which often forms homodimers or heterodimers with other bHLH proteins (Feller et al., 2011; Shen et al., 2016; Xing et al., 2018b). MYC TFs, belonging to bHLH family, possess a JAZ interaction domain (JID) in the N-terminal region, which differentiates MYC from other bHLH proteins (Kazan and Manners, 2013). The bHLH family plays an important part in regulating the biosynthesis of secondary metabolites such as the flavonoid pathway in *Arabidopsis thaliana* (Outchkourov et al., 2014), the iridoid pathway in *Catharanthus roseus* (Van Moerkercke et al., 2016), and the anthocyanin pathway in *Chrysanthemum morifolium* (Xiang et al., 2015).

Eight bHLH TFs have been reported to participate in the regulation of biosynthesis of active ingredients in *S. miltiorrhiza*, namely, SmMYC2, SmMYC2a, SmMYC2b, SmbHLH51, SmbHLH10, SmbHLH148, SmbHLH3, and SmbHLH37. MYC2 is a core gene TF in the plant and is responsive to jasmonates (Yang et al., 2017). Zhou et al. (2016) discovered that the overexpression of SmMYC2 could significantly increase the yields of phenolic acids by simultaneously up-regulating phenylpropanoid biosynthesis pathway and tyrosine biosynthesis pathway. However, SmMYC2a regulates phenolic acid biosynthetic pathway by binding with an E-box motif within promoters of SmCYP98A14 and SmHCT6, while SmMYC2b only binds with an E-box motif within promoters of SmCYP98A14. Zhang et al. (2020) overexpressed bHLH3 in *S. miltiorrhiza*, and contents of caffeic acid (CA), salvianolic acid B (Sal B), and rosmarinic acid (RA) were decreased by 50, 62, and 50%, respectively, compared with the control; in addition, the four tanshinone ingredients, the cryptotanshinone (CT), tanshinone I (T-I), tanshinone II A (T-II A), and dihydrotanshinone I (DT-I) decreased to 3, 14.48, 9, and 38% of the control, respectively. Interestingly, SmbHLH37, another bHLH TF of subfamily R like SmbHLH3, negatively regulates the biosynthesis of phenolic acids due to a dual effect, both by repressive binding to promoters of biosynthetic genes, and by a negative feedback loop on jasmonic acid accumulation (Du et al., 2018). Along with suppressing key enzyme genes of the biosynthetic pathway, SmbHLH37 antagonizes transcription activator SmMYC2 and can interact with SmJAZs. In addition, SmbHLH51 positively regulates phenolic acid through up-regulating many enzyme genes in the biosynthetic pathways (Wu et al., 2018). SmbHLH10 can directly bind to G-box within promotors of genes in the pathway, activate the expression of genes, and finally up-regulate tanshinones biosynthesis (Xing et al., 2018b). Xing et al. (2018a) found SmbHLH148 induced the accumulation of phenolic acids and tanshinones through activating virtually the whole biosynthetic pathway of phenolic acids and tanshinones.

### MYB Family

The MYB family is one of the largest TF families in plants and possess three repeats (R1, R2, and R3). These are classified into four groups based on the number of adjacent repeats: 1R (R1/2, R3-MYB), 2R (R2R3-MYB), 3R (R1R2R3-MYB), and 4R (harboring four R1/R2-like) (Liu et al., 2015). The MYB family is known to participate in the regulation of primary metabolism, secondary metabolism, and plant development (Dubos et al., 2010).

It has been suggested that subgroup 4 of MYB family has a negative effect on the accumulation of phenylpropanoid metabolites and acts as transcriptional repressors of phenylpropanoid pathway by suppressing transcription of key enzymes (Zhang et al., 2013). Zhang et al. (2020) found that SmMYB39, a MYB TF in subgroup 4, acts as a repressor in the rosmarinic acid pathway. The transcripts and enzyme activities of C4H and TAT, two key enzyme genes, were all down-regulated by SmMYB39. Deng et al. (2020) found SmMYB2, which activated the expression and promotion of salvianolic acid accumulation through binding to the MBS1/MBS2/MRE elements within the promoter CYP98A14. The three MYBs belonging to subgroups 20, SmMYB9b, and SmMYB98b act as direct activators in tanshinone biosynthesis (Li S. et al., 2018; Xing et al., 2018a), while SmMYB98 can promote both tanshinone and phenolic acid accumulation (Hao et al., 2020). Ding et al. (2017) found SmMYB36, a novel member of R2R3-MYB in evolution, or SmMYB36-bHLH complexes could up-regulate tanshinone biosynthesis but inhibit phenylpropanoid biosynthesis in *S. miltiorrhiza* hairy roots. Moreover, SmMYB36 can not only influence secondary metabolism but also regulate primary metabolism and may be a potential tool to alter metabolic flux. Overexpression or suppressing-expression of SmMYB111 can up-regulate or down-regulate, respectively, the production of Sal B, and Li S. et al. (2018) speculated that SmTTG1-SmMYB111-SmbHLH51, a ternary transcription complex, may act as a positive regulator of the phenolic acid pathway. SmMYB1 promotes phenolic acid biosynthesis by activating the expression of CYP98A14. Interestingly, the interaction between SmMYB1 and SmMYC2 additively activates the CYP98A14 promoter (Zhou et al., 2021).

### AP2/ERF Family

AP2/ERF proteins are also one of the largest families of TFs in the plant (Ji et al., 2016) and consist of 40–70 conserved amino acids (Xie et al., 2019). They are identified by an APETALA2 (AP2)/Ethylene Responsive Element Binding Factor (EREB) domain and are classified into four families: AP2, ERF, RAV, and DREB (Yamasaki et al., 2013). The AP2/ERFs have been reported to participate in secondary metabolism. For example, a novel AP2/ERF, Ii049, regulates lignan biosynthesis in *Isatis indigotica* (Ma et al., 2017). In addition, AaERF1 positively regulates artemisinin biosynthesis genes in *Artemisia annua* (Xiang et al., 2019).

Four AP2/ERFs in *S. miltiorrhiza* have been studied to regulate the biosynthesis of tanshinones and phenolic acids. Sun et al. (2019) found that the overexpression of SmERF115 reduced the yield of tanshinones but increased the yield of phenolic acids, and it is speculated that SmERF115 controlled the biosynthesis of phenolic acids mainly through regulating the expression of SmRAS1. In contrast, SmERF1L1 inhibits the biosynthesis of phenolic acids but promotes the biosynthesis of tanshinones, suggesting that a balance may exist between biosynthesis of phenolic acid and tanshinone in *S. miltiorrhiza* (Huang et al., 2019). In addition, SmERF128 and SmERF6 can also positively regulate diterpenoid tanshinone biosynthesis in *S. miltiorrhiza*. SmERF128 activated the expression of SmCPS1, SmKSL1, and SmCYP76AH1, while SmERF6 only recognized the GCC-box of SmCPS1 and SmKSL1, respectively (Bai et al., 2018; Zhang et al., 2019).

### Other Families

Moreover, three GRAS TFs, two WRKY TFs, one AREB, one LBD, and one JAZ TF have also been identified to regulate active ingredients in *S. miltiorrhiza*.

GRAS TFs possess a C-terminal and comprise five conserved subdomains: LRI, VHIID, LRII, PFYRE, and SAW (Pysh et al., 1999; Hofmann, 2016). SmGRAS1, SmGRAS2, and SmGRAS3, all GRAS, are reported to influence tanshinone biosynthesis in *S. miltiorrhiza*, as positive regulators. Interestingly, SmGRAS2 may regulate the tanshinones biosynthesis through interacting with SmGRAS1, while SmGRAS1 and SmGRAS3 directly regulate the biosynthesis of tanshinones by activating SmKSL1 (Li et al., 2019, 2020).

The WRKY family is a large TF family present in flowering plants and can regulate secondary metabolite biosynthesis (Yu et al., 2018) and interact with W-box (TTGACC/T) within the promoter of genes (Phukan et al., 2016). SmWRKY1 plays a role in the regulation of tanshinones biosynthesis and acts as a positive regulator through activating SmDXR in the MEP pathway, while SmWRKY2 positively regulates tanshinones through activating SmCPS in the downstream pathway (Cao et al., 2018; Deng et al., 2019).

The LBD proteins consists of approximately 100 amino acids with the N-terminal lateral organ boundaries (LOB) domain (Lu et al., 2020). Transgenic plants overexpressing SmLBD50 inhibit the synthesis of total phenolic acids in *S. miltiorrhiza*. It was speculated that LBD TFs may locate downstream in the JA signaling pathway and serve as the downstream gene of bHLH and MYB TFs, which play important parts in the biosynthesis of secondary metabolites in *S. miltiorrhiza* (Lu et al., 2020).

JAZ TF family can repress JA-dependent responses (Pauwels and Goossens, 2011), and Pei et al. (2018) found that SmJAZ8, which acted as a core repressor regulating JA-induced phenolic acid and tanshinone biosynthesis in *S. miltiorrhiza* hairy roots, might directly interact with SmMYC2a and suppress its activity. SmAREB1 is a special TF, and the transcriptional activation assay showed it has no activity, but the SmSnRK2.6 protein interacts with the SmAREB1 protein and activates its transcription to positively regulate phenolic acid biosynthesis (Jia et al., 2017).

## Conclusion and Future Perspective

*S. miltiorrhiza* can be used for the prevention of vascular diseases, especially atherosclerosis and cardiac diseases, for example, myocardial infarction, myocardial ischemia/reperfusion injury, cardiac fibrosis, cardiac hypertrophy, and arrhythmia (Li Z.M. et al., 2018). Phenolic acids and tanshinones are the major active ingredients in *S. miltiorrhiza*. A large number of enzyme-coding genes in phenolic acid and tanshinone biosynthetic pathways have been over-expressed or down-regulated to enhance the production of these compounds. Recently, more attention has been focused on TFs, which can activate or inhibit the multiple genes involved in one or more biosynthetic pathways. In this review we have discussed the potential and current limitations of the use of TFs for improving the production yield of secondary metabolites.

To date, many TFs are hypothesized to regulate tanshinones and phenolic acids. The key TF candidates are screened through the response of exogenous inductors, the distributions of specific expression, and the homology with other TFs studied in other plants (Li et al., 2015; Yu et al., 2018; Zhang et al., 2018). However, only a few TFs have been experimentally proven to participate in biosynthetic regulation. We hope more experimental pieces of evidence can be offered, so that more reliable and efficient TFs could be found, and we propose that more experiments should be performed to verify the function of TFs. Moreover, although there are a large number of researches on the biosynthesis of phenolic acids and tanshinones, it has not been clear which special enzyme plays a part for some reactions. And it impedes the study of the mechanism in which TFs act.

Jia et al. (2017) found that SmAREB1 promoted greater metabolic flux to the phenolic acid-branched pathway by interacting with SmSnRK2.6, a protein kinase; however, more upstream factors of TFs in *S. miltiorrhiza* remain elusive. Protein kinases are common regulators of TFs. In addition, exogenous plant hormones, biological stresses, and abiotic stresses can influence the expression of TFs, but little is known about the specific mechanism. The deeper study of this can make it cheaper and more convenient to regulate TFs, so as to make the regulation of plant secondary metabolite biosynthesis easier.

Some TFs can display a dual action and can regulate two pathways simultaneously. Many TFs have been found to bind sites on the promoter regions of both flavonoid and artemisinin genes in *A. annua*. Phenolic acids and tanshinones are two valuable pharmaceutical secondary metabolites in *S. miltiorrhiza*. SmMYC2a/b and SmMYB98 have been found to positively regulate biosynthetic pathways of phenolic acid and tanshinone simultaneously. Therefore, parallel transcriptional regulation of phenolic acid and tanshinone biosynthesis deserves further study.

Once the biosynthetic regulation of active ingredients by TFs in *S. miltiorrhiza* has been clearly understood, its clinical application will become more efficient. Furthermore, the knowledge obtained during studies with this model medicinal plant can then be extended to other complex medicinal plants, thus laying a foundation for the clinical application of medicinal plants.

## Author Contributions

SW and BZ collected documents and prepared the manuscript. LZ and LQ arranged the outline and organized the manuscript. TH and KR investigated and modified the manuscript. All authors were involved in conceptualization of the manuscript and writing of the manuscript, read and approved the manuscript.

## Conflict of Interest

The authors declare that the research was conducted in the absence of any commercial or financial relationships that could be construed as a potential conflict of interest.
